# Synthesis of a Dual Functional Anti-MDR Tumor Agent PH II-7 with Elucidations of Anti-Tumor Effects and Mechanisms

**DOI:** 10.1371/journal.pone.0032782

**Published:** 2012-03-05

**Authors:** Ye Su, Xin Cheng, Yaohong Tan, Yunhui Hu, Yuan Zhou, Juanni Liu, Yuanfu Xu, Yinliang Xie, Caiyun Wang, Yingdai Gao, Jianxiang Wang, Tao Cheng, Chunzheng Yang, Dongsheng Xiong, Hua Miao

**Affiliations:** 1 State Key Laboratory of Experimental Hematology, Department of Pharmacy, Institute of Hematology & Hospital of Blood Diseases, Chinese Academy of Medical Sciences & Peking Union Medical College, Tianjin, People's Republic of China; 2 Laboratory of Molecular Carcinogenesis and Targeted Therapy for Cancer, State Key Laboratory of Biomembrane and Membrane Biotechnology, Institute of Zoology, Chinese Academy of Sciences, Beijing, China; 3 Matthew Mailing Centre for Translational Transplant Studies, Department of Medicine, University of Western Ontario, Ontario, Canada; 4 Chemical Biology Program, Broad Institute of Harvard and Massachusetts Institute of Technology, Cambridge, Massachusetts, United States of America; University of Helsinki, Finland

## Abstract

Multidrug resistance mediated by P-glycoprotein in cancer cells has been a major issue that cripples the efficacy of chemotherapy agents. Aimed for improved efficacy against resistant cancer cells, we designed and synthesized 25 oxindole derivatives based on indirubin by structure-activity relationship analysis. The most potent one was named PH II-7, which was effective against 18 cancer cell lines and 5 resistant cell lines in MTT assay. It also significantly inhibited the resistant xenograft tumor growth in mouse model. In cell cycle assay and apoptosis assay conducted with flow cytometry, PH II-7 induced S phase cell cycle arrest and apoptosis even in resistant cells. Consistently revealed by real-time PCR, it modulates the expression of genes related to the cell cycle and apoptosis in these cells, which may contributes to its efficacy against them. By side-chain modification and FITC-labeling of PH II-7, we were able to show with confocal microscopy that not only it was not pumped by P-glycoprotein, it also attenuated the efflux of Adriamycin by P-glycoprotein in MDR tumor cells. Real-time PCR and western blot analysis showed that PH II-7 down-regulated MDR1 gene via protein kinase C alpha (PKCA) pathway, with c-FOS and c-JUN as possible mediators. Taken together, PH II-7 is a dual-functional compound that features both the cytotoxicity against cancer cells and the inhibitory effect on P-gp mediated drug efflux.

## Introduction

Clinical cancer treatments still rely heavily on chemotherapy, especially for metastatic cancers and hematological malignancies. The major problem that hinders the cytotoxic effect of chemotherapy is the energy-dependent active efflux of multiple drugs in certain cancer cells, a phenomenon initially reported in 1973 [1] as multi-drug resistance (MDR). P-glycoprotein(P-gp), which is a member of the ATP-binding cassettes(ABC), was subsequently revealed as a culprit [2]. Other ABC family members, e.g. ABCC1 [3]–[5], ABCG2 [6]–[8], have since been shown to affect drug efflux. Up to 28 of the total 48 human ABC genes are possibly related to MDR [9].

The P-gp represents the most well established mechanism for MDR, with many clinical chemotherapy drugs being its substrates. The importance of P-gp proposed a strong rationale for the inhibition strategy, hence three generations of P-gp inhibitors have been developed, but most of which were discontinued for pharmacological or pharmacokinetic side effects [10]–[15]. Other P-gp-targeting anti-MDR strategies include but not limited to: P-gp specific peptides [16] or antibodies [17], downregulation of MDR1 gene with transcriptional repressors [18], [19] or siRNAs [20], [21], novel agents that are not substrates of P-gp [22], [23], or encapsulation of chemo-agents to evade P-gp efflux [24].

The traditional Chinese medicine has a large pharmacopoeia that includes over 5500 natural sources, 82.8% of which are plants. These substances form the basis of 100,000 to 500,000 prescriptions and may be a good source for the discovery of new antitumor compounds [25]. Our lab identified indirubin as the active agent of Danggui Longhui Wan (an 11-component, effective recipe against chronic myeloid leukemia) [26]–[31]. Hoessel et al. reported indirubin as a potent cyclin-dependent kinase inhibitor and synthesized indirubin-3-monoxime [32], which arrests the cell cycle in the G2/M phase and inhibits the proliferation of a wide range of cells. Based on the template of indirubin, we designed and synthesized a series of oxindole derivatives; MTT assay and SAR (structure-activity relationship) study were used to screen for the most effective compound, through which we identified PH II-7, which effectively kills sensitive and multi-drug resistant tumor cells *in vitro* and *in vivo*.

## Results

### SAR analysis based development of PH II-7

**Figure 1:**
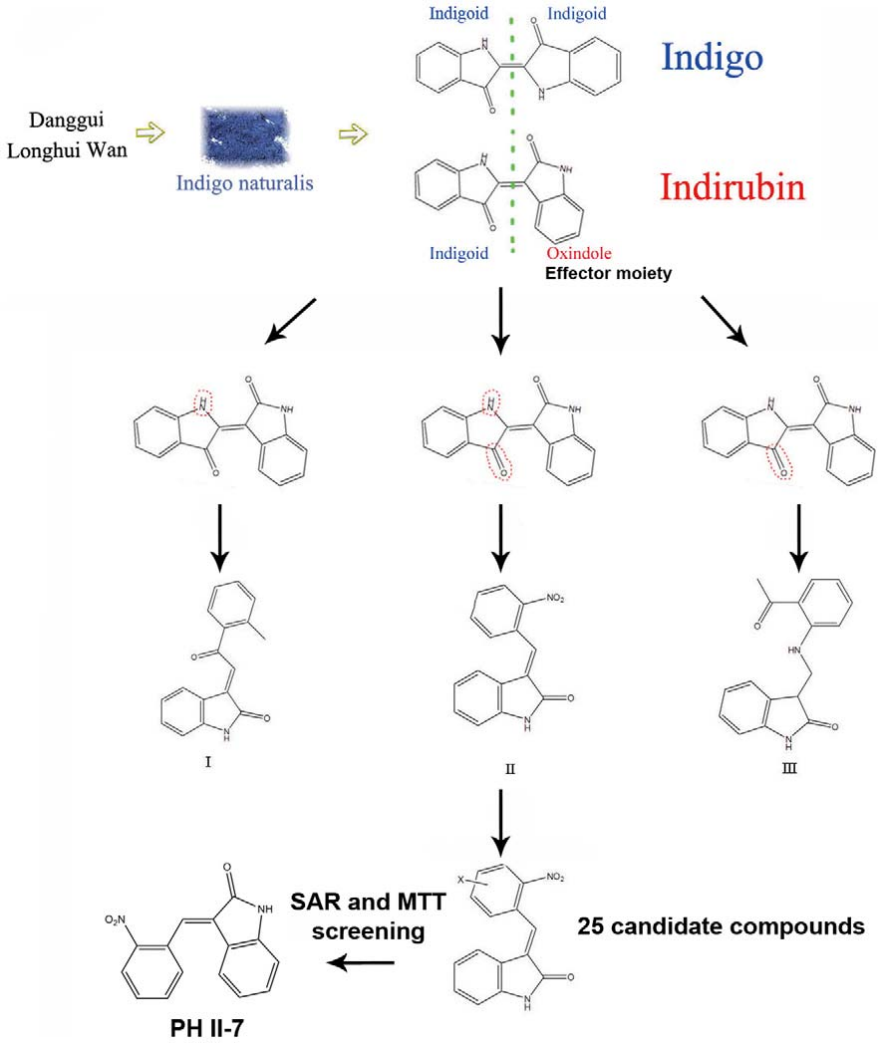
Schematic illustration of the process that leads to the discovery of PH II-7. From Danggui Longhui Wan to Indigo naturalis, then to indirubin, the anti-cancer bioactivity was finally attributed to oxindole, the effector moiety of indirubin, which was kept intact in the further modifications that lead to 3 kinds of compounds. Based on compound II, 25 candidates were generated by different modifications, among them PH II-7 was finally screened out by structure-activity relationship analysis and MTT assay.

Indirubin is composed of two structural units connected by a 2-3′ δ-bond, one is an indigoid and the other one is an indigoid isomer (Fig. 1). Indigo, with no bioactivity for tumor inhibition, is composed of two identical indigoid structural units connected by a 2-2′ δ-bond, indicating the bioactivity does not come with the indigoid, but with the indigoid isomer. We kept the indigoid isomer intact and modified the indigoid to generate three derivatives: I, II, and III. Through SAR and MTT analysis, we screened out compound II. Based on it we then synthesized 25 derivatives (Table S1) , among which PH II-7 was recognized as the most potent by further SAR analysis and MTT assay (Table S1,Fig. 1).

### PH II-7 shows potent cytotoxicity against both sensitive and MDR tumor cell lines

**Figure 2:**
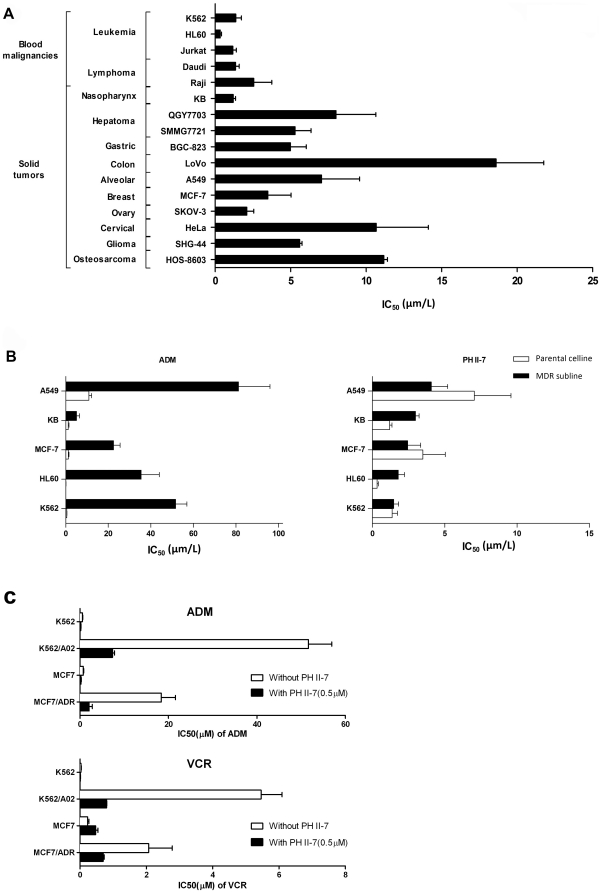
Efficacy of PH II-7 in cancer cell lines. (**A**) **IC50 value of PH II-7 in various human cancer cell lines.** IC50 values (mean ± SD, µM) were calculated from 3 independent experiments. (B) Cytotoxicity comparison between PH II-7 and ADM in five pairs of human tumor cell lines and their MDR sublines. IC50 values (mean ± SD, µM) were calculated from 3 independent experiments. The 5 MDR sublines are A549^DDP^, KB/v200, MCF-7/ADR, HL60/ADR, and K562/A02 respectively. (C) The IC50 values(mean ± SD, µM, calculated from 3 independent experiments) of Adriamycin (ADM, upper panel) and Vincristine (VCR, lower panel) in K562, K562/A02, MCF7, MCF7/ADR cells, with or without PH II-7 (0.5 µM).

Wth MTT assay, We tested PH II-7 against 18 human cancer cells lines (Fig. 2A), including 5 pairs of MDR sublines and their parental cell lines (Fig. 2B). PH II-7 showed universal potency for all of the tested cell lines; the effect was especially notable for MDR cells which defy most conventional chemotherapeutic agents. The IC50 values of Adriamycin in the CML cell line K562/A02, the AML cell line HL60/ADR, and the breast cancer cell line MCF7/ADR, are 93.93, 377.13 and 17.88 times as resistant, respectively, compared with their parental cell lines. However, with PH II-7, the differences only ranged from 0.7 to 5.3 folds. When combined with PH II-7 (0.5 µM), the efficacy of ADM against MDR cancer cell lines (K562/A02, MCF7/ADR) was greatly augmented, the same combinatorial effect was also observed with another chemotherapy agent Vincristine (VCR) which, like ADM, is also a substrate of P-gp (Fig. 2C).

### *In vivo* anti-tumor activity of PH II-7

**Figure 3:**
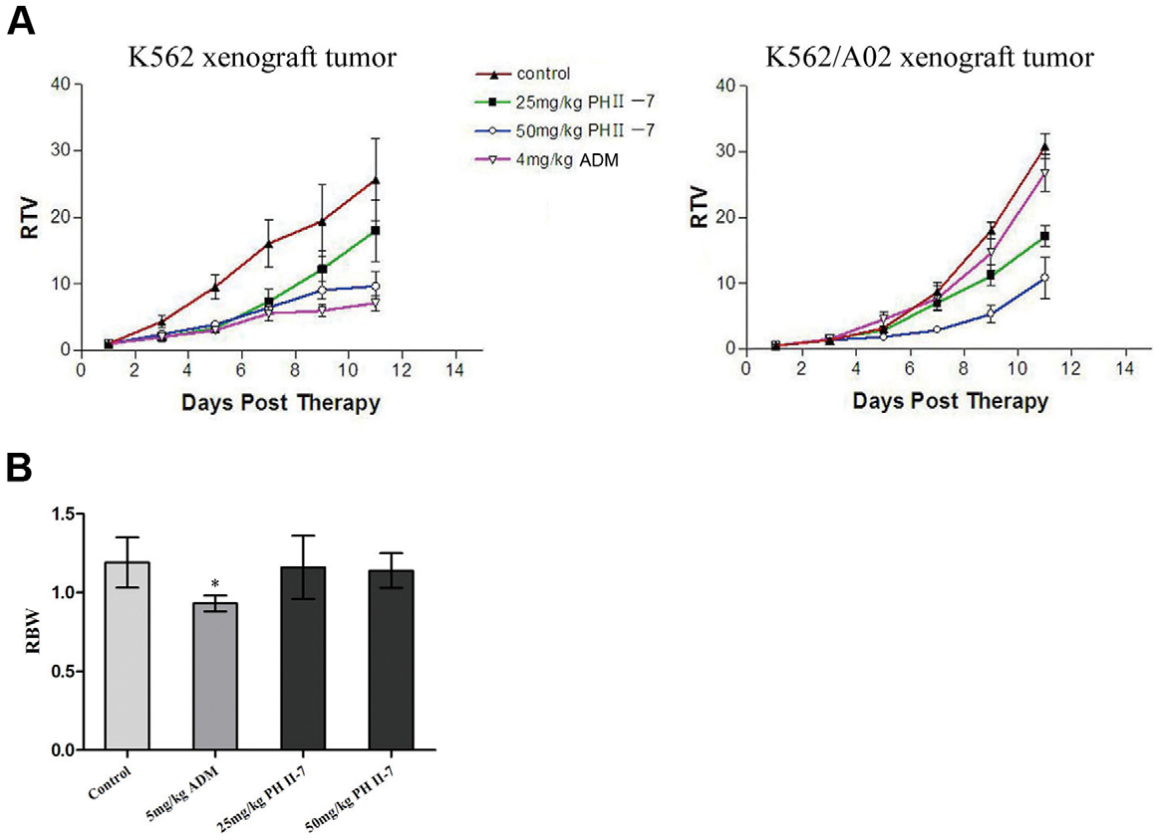
Efficacy of PH II-7 in K562 and K562/A02 xenograft tumors. (A) K562 and K562/A02 xenograft tumors were treated with PBS(control), 25 mg/kg PH II-7, 50 mg/kg PH II-7, 4 mg/kg ADM, separately. The relative tumor volumes (RTV) were measured and calculated over 11 days (n = 5). (B) Relative Body Weight (RBW) of each group (n = 5) (* : P<0.05).

Relative Tumor Volumes (RTV) of K562 and K562/A02 xenograft tumors treated with ADM or PH II-7 were calculated (Fig. 3A). ADM (5 mg/ml) exhibited an inhibitory rate of 70% on K562 cell xenograft tumors, but only 8.8% on K562/A02 cell xenograft tumors. The latter substantially resisted ADM treatment. Meantime, 25 mg/ml of PH II-7 inhibited K562 and K562/A02 xenograft tumors by 65.27% (P<0.05) and 51.94% (P<0.05), respectively, and no substantial resistance was observed. In all of the treated groups, PH II-7 was well tolerated; without considerable change in Relative Body Weight (RBW) even at the highest dose (5% increase, P>0.05) (Fig. 3B). While ADM was generally ineffective, PH II-7 substantially inhibited MDR xenograft tumor growth.

### PH II-7 induces apoptosis in both sensitive and resistant cell lines

**Figure 4:**
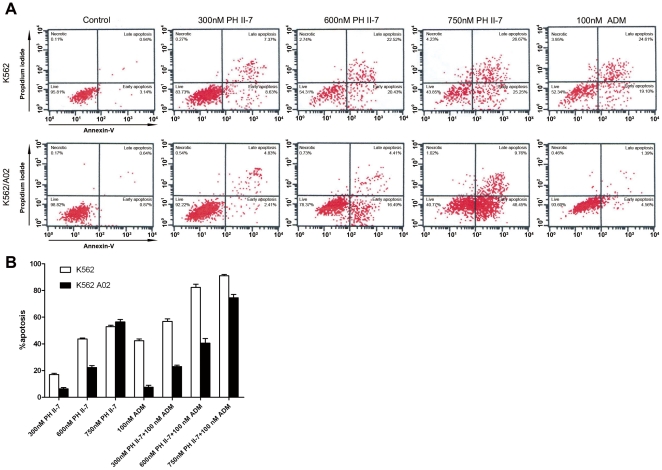
PH II-7 induces apoptosis in K562 and K562/A02 cells. (A) Induction of apoptosis by PH II-7 and ADM (both treated for 24 hours, concentrations indicated in the figure) in K562 or K562/A02 cells. (B) The induction of apoptosis in K562 and K562/A02 cells by different concentrations of PH II-7(300 nM, 600 nM, 700 nM), either alone or in combination with ADM(100 nM).

A series of concentrations (300 nM, 600 nM, and 750 nM) of PH II-7 induced apoptosis in K562 and K562/A02 cells (Fig. 4A). The percentage of cells in the early phase of apoptosis increased with the concentration of PH II-7 and there was no considerable difference in the PH II-7 dose/effect ratio between the two cell lines. ADM (100 nM) showed about the same apoptosis-inducing effect as 0.6 µM PH II-7 did in K562 cells, but failed to do so in K562/A02 cells(Fig. 4A). When ADM is used in combination with PH II-7, the pro-apoptotic effect was enhanced in a PH II-7 dose-dependent manner (Fig. 4B).

### PH II-7 induces cell cycle arrest in both sensitive and resistant cell lines

**Figure 5:**
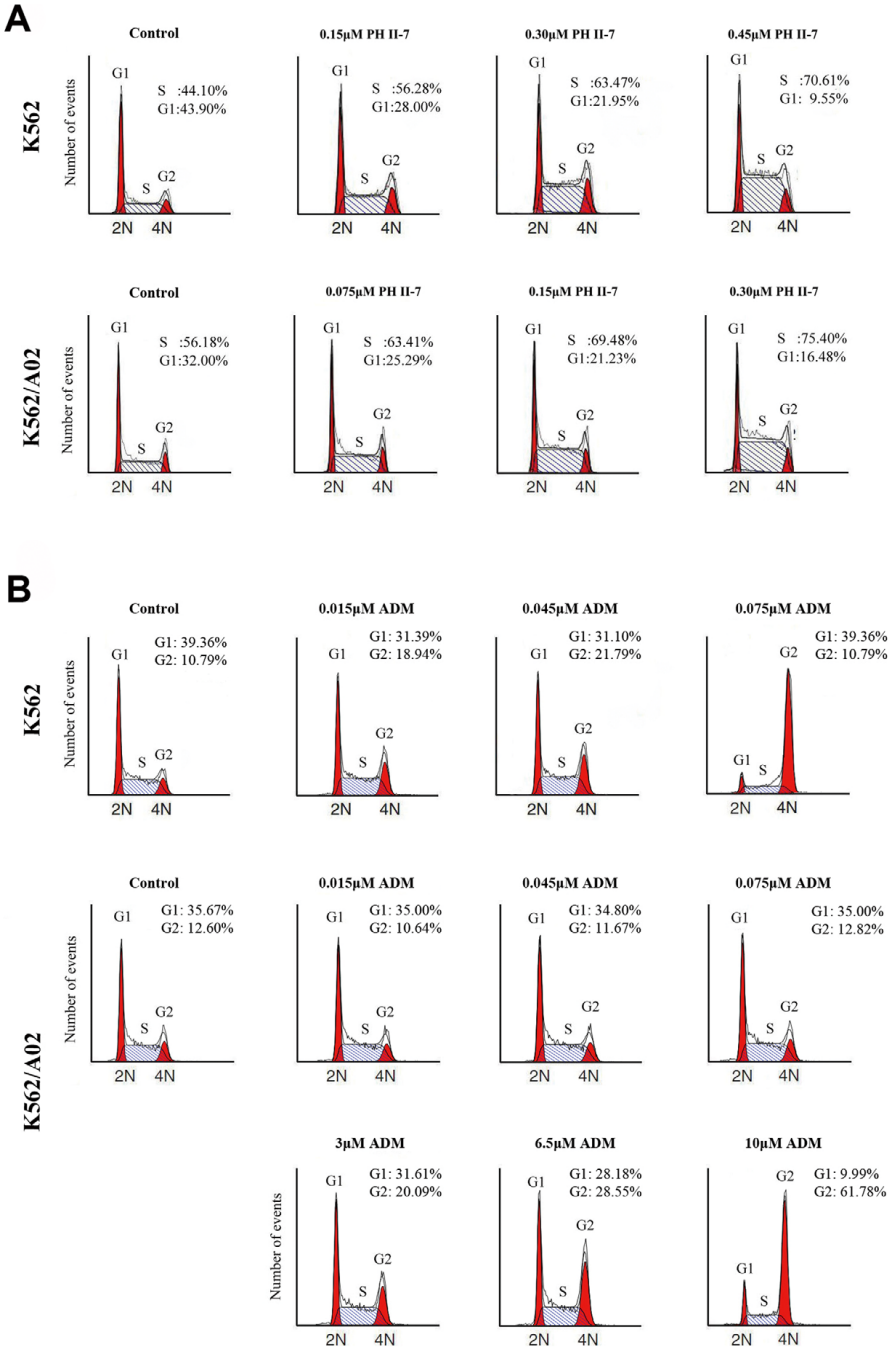
PH II-7 induces S phase cell cycle arrest in K562 and K562/A02 cells. (A) PH II-7 induces cell cycle S phase arrest in a concentration-dependent manner in K562 and K562/A02 cells, the cells were treated with the indicated concentrations of PH II-7 for 24 hours. (B) ADM induces cell cycle S-phase arrest in a concentration-dependent manner in K562 and K562/A02 cells; the cells were treated with the indicated concentration of ADM for 24 hours. The number of events is plotted against fluorescence intensity (DNA content).

we quantitatively evaluated the cell cycle arrest induced by PH II-7. The result (Fig. 5A) shows that PH II-7 caused a substantial increase in the percentage of S phase cells (from 44.10% to 70.61% and from 56.18% to 75.40%, in K562 and K562/A02 cells respectively), this contrasts with the inability of ADM to induce cycle arrest in K562/A02 at the dose effective to K562 cells (Fig. 5B). Furthermore, the percentage of cells inhibited in S phase increased with the concentration of PH II-7.

### CLSM imaging of labeled PH II-7 and ADM intracellular distribution

**Figure 6:**
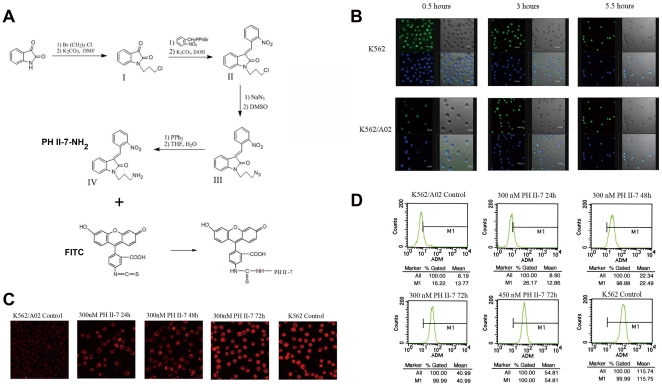
The intracellular distribution of PH II-7 in K562 and K562/A02 cells and its regulation of intracellular ADM concentration in K562/A02 cells. (A) Synthesis of 3-nitrobenzylidene-1-(3-aminopropyl) indoline-2-one (PH II-7-NH_2_), and the conjugation of it with FITC. (B)The intracellular locations of PH II-7 in K562 and K562/A02 cells at 0.5 hour, 3 hours, and 5.5 hours. Green dots indicate the FITC-labeled PH II-7 derivative. Blue dots indicate cell nuclei. (C) Adriamycin intracellular concentration augmented in a time dependent manner in PH II-7 treated K562/A02 cells. (D) Modulation of ADM intracellular concentration by PH II-7 in K562/A02 cells measured by flow cytometry. The X-axis represents intracellular ADM concentration, while the Y-axis represents the cell count.

We made side chain decoration on PH II-7 (3-nitrobenzylidene-1-(3-aminopropyl) indoline-2-one, or PH II-7-NH_2_, method described in supplement), to conjugate it with Fluorescein isothiocyanate (FITC) (Fig. 6A). CLSM imaging illustrated that FITC-labeled PH II-7 aggregated in the nuclei of both the K562 and the K562/A02 cells in a time-dependent manner. However, there was no considerable change in the overall intracellular concentration of PH II-7 between the two cell lines at the three time points (Fig. 6B). In comparison, the intracellular concentration of ADM more sharply decreased in the K562/A02 cells than in the K562 cells (Fig. 6C). The decrease could be reversed (in a time-dependent manner) by the administration of PH II-7 at a very low concentration (300 nM) (Fig. 6C).

### Measurement of ADM intracellular accumulation and efflux by Flow Cytometry

We quantitatively defined the effect of PH II-7 on drug accumulation and efflux by flow cytometry. The intracellular drug concentration increase was in a PH II-7 dose-dependent manner (Fig. 6D), indicated that PH II-7 partially inhibited the drug efflux in K562/A02 cells.

### PH II-7's effect on genome wide mRNA profile

We investigated changes of the genome wide mRNA profiles in cells treated with PH II-7. The cRNA was generated from isolated total RNA samples from K562 and K562/A02 cells treated for 48 hours with 200 nM of PH II-7; cRNA was labeled, hybridized and scanned according to standard Affymetrix protocols. Affymetrix Human U133 Plus 2.0 Arrays, which contain more than 54,000 probe sets, were used for each RNA sample. We determined alterations in transcription levels by comparing our information with data derived from the same cell lines treated with PBS (as a control) under identical conditions. PH II-7-responsive genes were defined as those whose expression levels were up-regulated or down-regulated by PH II-7 by more than two folds. These genes were assigned to six groups: those up-regulated by PH II-7 in both sublines, in K562/A02 only, or in K562 only and those down-regulated by PH II-7 in both sublines, in K562/A02 only, or in K562 only.

**Figure 7:**
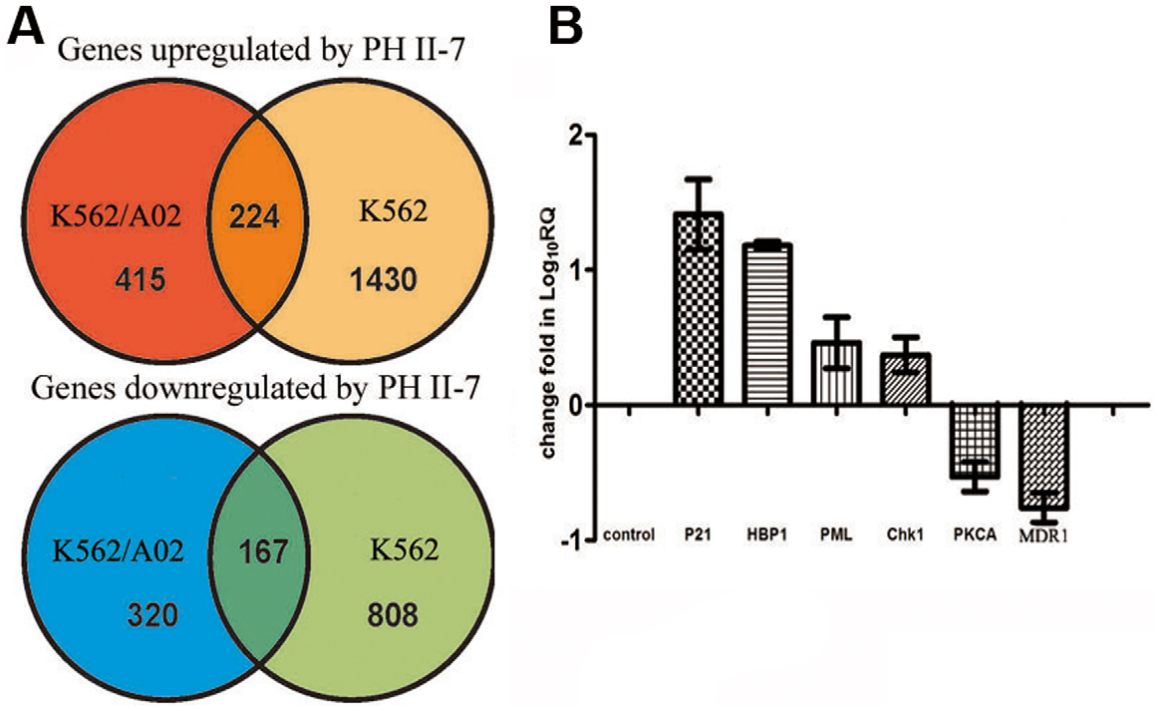
Regulation of gene expression by PH II-7 in K562 and K562/A02 cells. The K562 and K562/A02 cells were treated with PBS or PH II-7 (200 nM) for 48 hours, (A)Venn diagrams showing numbers of PH II-7-responsive genes in K562 and K562/A02 cells. PH II-7-responsive genes were defined as genes whose expression levels were up-regulated or down-regulated by PH II-7 (200 nM, 48 hours) by more than 2-fold and were assigned to 6 groups: up-regulated by PH II-7 in both cell lines, K562/A02 only, and K562 only; down-regulated by PH II-7 in both cell lines, K562/A02 only, and K562 only. (B) Genes showed significant expression level change compared with control (P<0.05) in further validation by RT-qPCR. Data represent results from at least 4 separate experiments.

We found a series of PH II-7 responsive genes including cell cycle related genes (such as CHK1, P21, and HBP1), apoptosis related genes (such as PML), MDR related genes (such as MDR1, PKCA) (Fig. 7A), these are put to further screening and validation with real-time PCR. Gene ontology analysis revealed over-represented functional classes of genes that are either up-regulated or down-regulated by PH II-7 (Table 1).

**Table 1:** Gene Ontology analysis, over-represented functional classes of genes.

Up-regulated by PH II-7	Down-regulated by PH II-7
Cellular process (P<0.003)	Cell cycle (P<0.02)
Apoptosis (P<0.03)	Cellular physiological process (P<0.03)
Programmed cell death (P<0.03)	Cytoskeleton (P<0.006)
	Phosphatidylinositol signaling system (P<0.05)

### Relative quantification of interested genes by real-time PCR

Expression variations of some important genes are further validated and qualified by real-time relative quantitative PCR (RT-qPCR). The K562 and K562/A02 cells were treated with PBS or PH II-7 (200 nM) for 48 hours, and the total RNA was extracted, reverse-transcribed to cDNA, which was used for the real-time relative quantitative PCR. Several cell cycle related genes CHK1, P21, and HBP, and the apoptosis related gene PML, are significantly up-regulated by PH II-7 treatment, and two MDR related genes PKCA and MDR1, were significantly down-regulated (P<0.05, n = 4) (Fig. 7B).

### PH II-7 reduces MDR1/P-gp expression through PKCA related pathway

**Figure 8:**
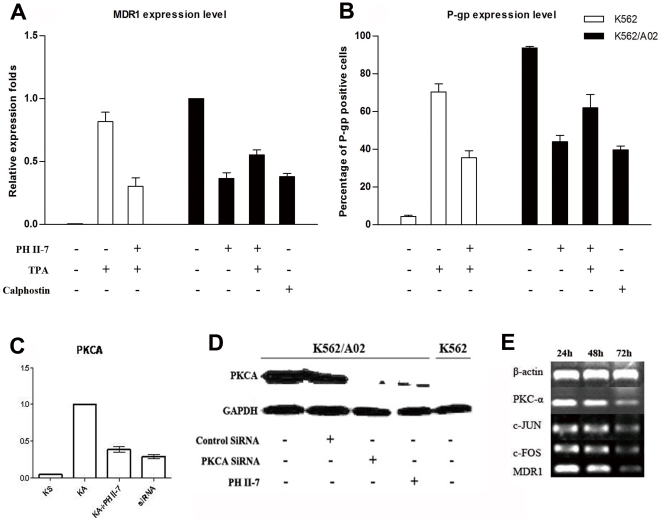
PH II-7 regulates MDR1/P-gp expression through a PKCA pathway. (A) The expression of MDR1 in K562 and K562/A02 cells with or without the treatments of either PH II-7 (200 nM), TPA (100 nM), Calphostin (1 µM), or their different combinations, detected by RT-qPCR. Expression levels are normalized to that of untreated K562/A02 (as 1) (n = 4). (B) The surface expression of MDR1 in K562 and K562/A02 cells with or without treatments of either PH II-7, TPA, Calphostin, or their different combinations, detected by flow cytometry(n = 4). (C) mRNA expression of PKCA in K562, K562/A02, K562/A02 treated with PH II-7(200 nM), K562/A02 treated with siRNA targeting PKCA, detected by RT-qPCR (n = 4). (D) Western-blot, protein level of PKCA in K562/A02, K562/A02 treated with control siRNA(scramble), K562/A02 treated with siRNA targeting PKCA, K562/A02 treated with PH II-7(200 nM), K562. GAPDH was used as an internal control. (E) Transfection of K562/A02 cells with PKCA-targeted siRNA, resulted in mRNA expression knock down in PKCA, MDR1, and also two transcriptors downstream of PKCA, c-JUN, c-FOS.

We used the PKCA activator, 12-O-Tetradecanoylphorbol-13-acetate(TPA), and the PKCA inhibitor, Calphostin. The naïve expression of MDR1/P-gp is critically higher in K562/A02 than in K562. TPA induces MDR1/P-gp expression in K562, which is significantly antagonized by PH II-7(P<0.05). As does Calphostin, PH II-7 reduces MDR1/P-gp expression in K562/A02, the effect is significantly antagonized by TPA (P<0.05). (Fig. 8A,B). The PKCA mRNA and protein levels, which are critically higher in K562/A02 than in K562, are significantly reduced by either PH II-7 (P<0.05) or PKCA siRNA (P<0.05) (Fig. 8C,D). Interestingly, two transcription factors, c-JUN and c-FOS, were also downregulated by PKCA siRNA, along with PKCA and MDR1 (Fig. 8E).

## Discussion

Both inhibition of P-gp efflux function and downregulation of MDR1/P-gp expression are theoretically effective strategies to reverse MDR, and many dedicated P-gp inhibitors or dedicated MDR1 expression regulators have been developed, but their efficacies are still to be improved. Even with satisfying efficacy, they can only be supplements to classic chemotherapy agents, hence in extra, could cause undesirable drug-drug interactions, toxicities or pharmacokinetic side effects [10], [11], [13], [15].

Being a potent cancer cell-killing agent in the first place, PH II-7 is also effective in inhibiting P-gp efflux function and in reducing MDR1/P-gp expression, thus represents a promising lead compound in MDR cancer therapy.

Primarily, PH II-7 is a potent anti-tumor agent, especially against MDR tumor. PH II-7 has a broad antitumor spectrum (affecting at least 16 human cancer cell lines); it effectively inhibited the growth of all tested cancer cells at comparatively low concentrations (Fig. 2A). Compared to the chemotherapeutic agent ADM, PH II- 7 is especially potent against 5 pairs of human cancer cell lines and their corresponding MDR sublines that are highly resistant to ADM (Fig. 2B). The potency against MDR tumor cells indicates that PH II-7 could have different action modes compared with conventional chemotherapeutic agents. Furthermore, in combinatorial treatment, PH II-7(0.5 µM, much lower than IC50), significantly potentiates ADM and Vincristine (VCR) (Both are substrates of P-gp) against MDR cancer cells (Fig. 2C), Which suggests certain degree of re-sensitization of P-gp overexpressing MDR cells by PH II-7. Consistently, when ADM is only effective against the xenograft tumor of K562 but not that of K562/A02, PH II-7 is effective on both (Fig. 3A) at doses better tolerated than ADM, without significant host body weight loss (Fig. 3B).

The potency of PH II-7 against MDR cancer cells can at least be partially attributed to its ability to induce apoptosis and cell cycle arrest in MDR cells.

MDR cancer cells treated with chemotherapeutic agents develop apoptosis resistance or a reduced apoptosis response. Pallis reported that P-gp has a drug-independent role in the inhibition of in vitro apoptosis in AML blasts [33]. The modulation of cytokine efflux, signaling lipids and intracellular pH have all been suggested as ways by which P-gp may affect cellular resistance to apoptosis [34]. The Adriamycin selection of the human multiple myeloma cell line RPMI 8226 led to changes in gene expression that reduced the apoptotic response to death-inducing stimuli; this reduction contributes to the multidrug resistance phenotype [35]. ADM (100 nM) induced significant apoptosis in K562 cells but failed to do so in K562/A02 cells (Fig. 4A). Notably, apoptosis was induced in K562 and K562/A02 cells treated with PH II-7 despite the concentration variation (Fig. 4A), and the apoptosis resistance to ADM treatment can be reversed by PH II-7 dose dependently (Fig. 4B), which indicates that PH II-7 is capable of overcoming apoptosis resistance and inducing significant apoptosis in K562/A02 cells.

Resistance to cell cycle arrest is another way P-gp helps MDR cancer cells survive the conventional chemo-agents [36]. Previous studies have demonstrated that certain indirubin derivatives are capable of inducing cell cycle arrest in cancer cells [37]–[39]. Our data indicate that PH II-7 substantially inhibited the cell cycles of not only K562 but also K562/A02 cells in S phase (Fig. 5A,B). Physiologically, by ensuring that the onset of mitosis is dependent on the completion of DNA replication, the S phase checkpoint prevents the generation of aneuploid daughter cells that are not viable. MDR cells inhibited in S phase by PH II-7 are not able to enter G2 and M phases and are therefore prevented from undergoing mitosis and further proliferation.

Being a very potent anti-cancer agent itself, PH II-7 features yet another much-needed characteristic against MDR cancer cells: inhibition of P-gp mediated drug effux. K562/A02 cells overexpress P-gp; silencing of MDR1 by RNAi resulted in an attenuated MDR phenotype and increased drug efficacy [4]–[8]. CLSM imaging revealed a dramatic decrease of ADM retention in K562/A02 cells compared with K562 cells. However, the intracellular retention of FITC-labeled PH II-7 did not significantly vary between K562 and K562/A02 cells; the PH II-7 molecules redistributed into the nucleus over time, rather than being pumped out of the cells like ADM(Fig. 6B). Hence PH II-7 is not influenced by the drug efflux mediated MDR mechanism, quite possibly because it is not a substrate of P-gp.

The function of P-gp can be influenced by small molecule compounds [40]–[43]. K562/A02 cells that were pretreated with a very low concentration (<IC10) of PH II-7 showed attenuated drug efflux that was dependent on both time and concentration (Fig. 6C,D). This effect may at least partially contribute to the blunted P-gp funtion in cells treated with PH II-7. We found that PH II-7 reduced the expression level of MDR1, along with protein kinase C alpha (PKCA), a gene that is intimately related to MDR1 expression(Fig. 7B, Fig. 8A–D). Others reported that PKCA acts as a regulator of MDR1 expression, which is supported by the increased MDR1 expression in K562 cells in response to the PKCA agonist TPA [44], [45] and by the fact that the transfection of PKC-α antisense cDNA into MCF-7/ADR cells attenuates their MDR phenotype [46], [47]. The induction of MDR1 activity by PKCA in the presence of TPA can be attenuated by the PKC inhibitor GF 109203X [48]. Our data indicate that the reduction of MDR1/P-gp expression by PH II-7 is antagonized by PKCA activator TPA, while the induction of MDR1/P-gp expression by TPA is abrogated by PH II-7, which strongly recommends the involvement of PKCA in PH II-7's regulation of MDR1(Fig. 8A–C). Whereas in TPA-alone treated K562/A02 cells, since the PKCA is congenitally expressed at a high level, no significant difference in MDR1/Pgp expression was observed(data not shown). PH II-7 and Calphostin alike can reduce MDR1/P-gp expression(Fig. 8A,B), while PH II-7 and PKCA siRNA alike can reduce PKCA expression(Fig. 8C,D), which suggests that PH II-7 downregulates MDR1 by suppressing PKCA expression. Interestingly, two transcription factors c-FOS and c-JUN, which are downstream under regulation of PKCA [49]–[52], bind to AP-1 site as homodimer or heterodimer [53], [54]. An AP-1 element exists in the MDR1 promoter [55], [56], and numerous reports confirmed the regulation of MDR1 expression by c-FOS/c-JUN binding/activation of AP-1, but debates arise when it comes to ‘how’, some reported the regulation to be positive [55]–[58], while some believed it to be negative [59], [60]. The inconsistency could at least be partly attributed to the different cells and environments upon which these experiments were conducted. In K562/A02 cells, our data show that along with the knock-down of PKCA expression, the c-FOS/c-JUN and MDR1 expressions decreased simultaneously(Fig. 8E), strongly indicates a positive regulation of MDR1 by c-FOS/c-JUN. Therefore, with c-FOS/c-JUN as mediators that bridge the PKCA-to-MDR1 regulation, we propose the possible pathway as: PH II-7 suppresses PKCA expression, and in turn reduces the c-FOS/c-JUN expression and their binding/activation of AP-1 element in the MDR1 promoter region, thus inhibits MDR1/P-gp expression.

Besides PKCA, several other PH II-7 responsive genes revealed by RT-qPCR are possibly responsible for its efficacy, including PML, HBP1, CHK1 and P21. PH II-7 treatment in both K562 and K562/A02 cells induced expression of these genes (Fig. 7B). PML is essential for multiple apoptotic pathways. Analysis of mouse cells lacking PML has demonstrated that PML is involved in various apoptotic pathways. Hematopoietic cells and embryonic fibroblasts from PML nul,ll mice are resistant to a series of p53-dependent and p53-independent pro-apoptotic stimuli [61]. We believe that inhibition of PML expression by PH II-7 inevitably contributes to the circumvention of apoptosis resistance in K562/A02 cells. HBP1 is controlled by p38 mitogen-activated protein kinase pathway and regulates cell cycle progression via NADPH oxidase [62], [63]. CHK1 has been reported to be involved in S phase cell cycle arrest. The depletion of CHK1 resulted in the loss of S phase arrest upon incubation with topoisomerase I inhibitor SN38, which arrests cells in S phase [64]. CHK1 is also reported to be a an intra-S phase checkpoint, ensuring the integrity of the arrested replication fork, and CHK1 inhibitor causes global firing of replication origin [65]. Numerous reports indicate that p21 expression is under the positive regulation of PKCA [66], [67], the overexpression of p21 arrests cell cycle in S phase [68]. P21 interacts with E2F-1 to regulate cell progression to guard against unrestricted cell proliferation [69]. The cell cycle arrest by PH II-7 (Fig. 5A,B) may partially be attributed to the regulation of PH II-7 on CHK1 and p21. Thus, these mechanisms, aside from inhibiting and regulating P-gp, could also be exploited by PH II-7 to exert its efficacy against cancer cells, especially the resistant ones.

In conclusion, we used indirubin, the active component of the traditional Chinese medicine Danggui Longhui Wan, as a template to synthesize the oxindole derivative PH II-7. PH II-7 shows potent broad spectrum antitumor activity, especially against MDR tumor cells, both *in vitro* and *in vivo*. PH II-7 achieved a much higher intracellular concentration than other chemotherapeutic agents and significantly induced apoptosis and S phase cell cycle arrest in MDR tumor cells. These actions occurred through the altered expression of specific genes and resulted in the effective inhibition and termination of MDR tumor growth. In addition, *in vitro* and *in vivo* experiments show excellent tumor bearer tolerance. We further found that PH II-7 effectively inhibits the expression of MDR1 and impairs the drug efflux function of P-gp by reducing the expression level of PKCA. Our data suggest that PH II-7 is a promising lead compound for anti-MDR tumor drug development and may offer a new solution to the MDR phenomenon. Our method of discovering PH II-7 represents an effective paradigm of lead compound development based on traditional Chinese medicine.

## Materials and Methods

### Cell culture

Drug-selected cell lines overexpressing P-gp (K562/A02, MCF-7/ADR, KB/v200 and A549DDP) and MRP-1 (HL60/ADR) were cultured with Adriamycin (ADM), Vincristine or Cisplatin , as previously described [28]–[33]. K562/A02 cell line showed a significant (100-fold) increase in drug resistance. TPA (PKC activator, sigma) and Calphostin C (PKC inhibitor, sigma) were used for cell treatment in some experiments.

### Cytotoxicity Assay

Leukemia cells (2×10^4^/well) were seeded 96-well tissue culture plates (Coaster, Charlotte, NC) and treated with derivatives I, X-substitutes, PH II-7, Adriamycin or PH II-7 plus Adriamycin for 72 hours at 37°C in a 5% CO_2_ atmosphere. Twenty microliters of MTT (5 mg/mL) was added to each well and the plates were incubated for 4 hours, then 100 µl DMSO was added to each well. The absorbance value of the cells was read at 570 nm using a spectrophotometer (Model A-5082, SLT Lab Instrumenta, Grodig, Austria).

### Animals

Five to six weeks old female nude mice (BALB/C, nu/nu, body weight 16–18 g) were obtained from Vital River Laboratories. All animal studies were conducted according to protocols approved by the Animal Ethics Committee of the Institute of Hematology & Hospital of Blood Diseases, Chinese Academy of Medical Sciences & Peking Union Medical College, with the approval ID of AEC2009070202. All mice used in this study were bred and maintained in a specific pathogen-free environment.

### In vivo anti-tumor PH II-7

The mice were exposed to total body irradiation (400 rad) to suppress their residual immune systems and facilitate the establishment of xenografts. Twelve hours later, the mice were injected subcutaneously in the right flank with 2×10^7^ K562 and K562/A02 cells suspended in PBS. When the tumor volume reached 40 to 60 mm^3^, the animals were randomly assigned to 4 groups, each separately treated (i.p. TID) with 4 mg/kg ADM alone, 25 mg/kg PH II-7 alone, 50 mg/kg PH II-7 alone, or PBS (control). Tumor dimensions were measured using a linear caliper every 2 days and tumor volume (RTV) was calculated using the equation RTV (mm^3^) = a×b^2^/2, where a is the longest diameter and b is the shortest diameter. The relative body weight (RBW) was calculated by the equation RBW = Wt/Wo, where Wt is the body weight on day 11, Wo is the body weight on day 0.

### Apoptosis assay

K562 and K562/A02 cells were seeded in 6-well plates and exposed to different concentrations of PH II-7 (0.3 µM, 0.6 µM, or 0.75 µM) or ADM (100 nM) for 24 hours. To rule out the influence of PH II-7 cytotoxicity, the concentration was limited to no more than 0.75 µM. Samples were labeled using an annexin V-FITC and PI double-staining apoptosis detection kit purchased from Becton Dickinson (Mountain View, CA). Cells were assayed using flow cytometry.

### Cell cycle test

Logarithmically growing K562 and K562/A02 cells (5×10^5^) were seeded in 6-well tissue culture plates (Coaster, Charlotte, NC) and incubated with different concentrations of PH II-7 or ADM for 24 hours. Cell harvesting and staining were completed with a CycleTEST™ PLUS DNA Reagent Kit purchased from Becton Dickinson (Mountain View, CA). The percentage of cells in each cycle phase was calculated with Modfit software (Verity Software House, U.S).

### Flow cytometric measurement of intracellular ADM concentration

Cells were cultured in 24-well plates and exposed to different concentrations of PH II-7 for 24 hours, 48 hours and 72 hours. They were then incubated with ADM for 1 hour and their intracellular fluorescence was examined by flow cytometry.

### Side chain modulation, FITC labeling and intracellular localization of PH II-7

The names and synthetic protocols for the four compounds listed in Fig. 4A are as follows:

I 1-(3-chloropropyl) indoline-2,3-dione; the synthesis followed the method described by Torisawa.
II 3-nitrobenzylidene-1-(3-chloropropyl) indoline-2-one; the synthesis followed the method described by Messaoudi.
III 3-nitrobenzylidene-1-(3-azidopropyl) indoline-2-one; the synthesis followed the method described by Ortiz.
IV 3-nitrobenzylidene-1-(3-aminopropyl) indoline-2-one; the synthesis followed the method described by De Almeida.

To a stirred solution of 2.5 ml bicarbonate buffer (0.02 M, pH 9.3), PH II-7 amino-propyl-side chain derivative (4 mg) and FITC (4 mg) were added; the mixture was incubated at room temperature for 17 hours and then purified by thin-layer chromatography (acetidin∶ligroin, 10∶1, vol/vol). The mixture was evaporated under reduced pressure to yield the FITC labeled PH II-7 derivative as a maroon solid: R_f_ 0.1, yield 86%, mp 188–189°C, 1H NMR [CDCl3, 400 MHz]: δ = 0.91 (t, *J* = 7.2 Hz, 3H, CH3), 1.36–1.43 (m, 2H, CH2), 1.63–1.68 (m, 2H, CH2), 3.34 (d, *J* = 6.0 Hz, 2H, CH2), 7.13 (t, *J* = 8 Hz, 1H), 7.36 (d, *J* = 7.6 Hz, 1H), 7.58 (d, *J* = 7.6 Hz, 1H), 10.52 (s, 1H, NH). ESI-MS: (M-) m/z (%) = 282 (100); Anal. Calc'd for C_12_H_14_ClN_3_OS. (283.78): C, 50.79; H, 4.97; N, 14.81. Found: C, 50.52; H, 4.71; N, 14.80.

Compound IV and FITC (1∶1) mixture were incubated at room temperature for 17 hours and then bonded products were purified by thin-layer chromatography.

### Confocal Microscopy

Images of the numbered and subcultured cells in 6-well plates were obtained using a confocal fluorescence microscope (Leica, Nussloch, Germany). Adriamycin can be activated by 500–566 nm wavelength green excitation laser, and emits 620 nm wavelength red fluorescence [70]–[72], we used 544 nm wavelength excitation laser in this experiment. 488 nm/522 nm were set as the excitation and emission wavelengths of FITC labeled PH II-7 respectively.

### Genome profile analysis

The K562 and K562/A02 cells were treated with PBS or PH II-7 (200 nM) for 48 hours, and the total RNA was extracted, reversed transcribed to cDNA, then in vitro transcribed to cRNA, and hybridized to the HGU 133 plus 2 arrays, which were then scanned and analyzed using Affymetrix GeneChip Operating Software (GCOS) version 1.4. The GCOS algorithm analyzes data in the following ways (1) determine whether calls of individual genes are present or not by evaluating probe pairs for matching status; (2) normalize raw gene expression values and calculate their relative expression levels; and (3) statistically decide the relative gene expression variation. Transcripts with expression intensities lower than 100 in all samples were excluded since the extremely low signal level could be intertwined with the background noise, and the fold-changes tend to be distorted. Transcripts with an absent call in all samples were also excluded. Significantly expressed genes were defined as those with expression levels up-regulated or down-regulated by more than a factor of 2. Annotation analysis was conducted by R. Bioconductor, MeV.

### Gene ontology analysis

Gene ontology (GO) analysis was performed with the online tool of The Database for Annotation, Visualization and Integrated Discovery (DAVID) v6.7 [73], [74], url: http://david.abcc.ncifcrf.gov/.

### Real-time PCR

Real-time polymerase chain reaction (PCR) was used with GAPDH as the reference gene. The PCR reactions were done in a final volume of 20 µL containing cDNA synthesized from 2 µg total RNA (see above), 330 nM primers, and 10 µL SYBR-green PCR master mix (Applied Biosystems, Foster City, CA). Results from at least 4 separate experiments were calculated using the 7500 system Sequence Detection Software version 1.2.3 (Applied Biosystems).

### siRNA transfection

PKC-specific siRNA (Invitrogen) was transfected into leukemia cells using Lipofectamine 2000 according to the protocol recommended by the manufacturer. The effects of siRNA on PKCA and MDR1 mRNA expression were examined 72 hours after transfection by RT-PCR.

### Western blot analysis for PKCA

K562/A02 cells (5×10^5^/ml) were treated with PH II-7, TPA, Calphostin C or PKC-specific siRNA at 37°C for 72 hours. Harvested cell pellets were suspended in PBS and then lysed in lysis buffer (50 mM Tris-HCl (pH 8.0), 150 mM NaCl, 1% NP40, 0.5% sodium deoxycholate, 1 mM DTT, 0.1% SDS, 1 mM ethylene diamine tetraacetic acid, 100 mg/L PMSF, and 1 mg/L aprotinin). Equal amounts of cell lysate were separated on 12% Tris-glycine-SDS polyacrylamide gels and proteins were electroblotted onto nitrocellulose membranes. Proteins were identified using polyclonal antibodies for PKCA and GAPDH. Detection was performed using horseradish peroxidase-conjugated secondary antibody and Super Signal West Pico Chemiluminescent Substrate (Pierce Inc, Rockford, IL) according to the manufacturers' instructions.

### Flow cytometry analysis for P-gp

P-gp expression was analyzed by flow cytometry. Briefly, K562/A02 cells (5×10^5^/ml) were exposed to PH II-7, TPA, Calphostin C or PKCA-targeted siRNA at 37°C for 72 hours. Harvested cell pellets were resuspended in PBS. About 1×10^6^ cells were incubated with phycoerythrin–conjugated P-gp or polyclonal human IgG as a nonbinding control in a final volume of 0.2 mL. Incubation was continued for an additional 60 minutes on ice. After 2 washes with serum-free medium, cells were analyzed using flow cytometry.

## Supporting Information

Table S1 IC50 values of the derivatives of PH II-7 in various human tumor cell lines.
(DOC)
