# Supplementary material for: Synthesis of a Dual Functional Anti-MDR Tumor Agent PH II-7 with Elucidations of Anti-Tumor Effects and Mechanisms
Source: PLoS One. 2012 Mar 5;7(3):e32782. doi: 10.1371/journal.pone.0032782 (PMC3293869; doi:10.1371/journal.pone.0032782)
Supplement: Table S1 — IC50 values of the derivatives of PH II-7 in various human tumor cell lines. (DOC) [file pone.0032782.s001.doc]

**Table S1** IC50 values of the derivatives of PH II-7 in various human tumor cell lines.

|  | Structure | MW | 72h IC50 L1210 10^-7^M | 72h IC50 K562 10^-7^M | 72h IC50 K562/A02 10^-7^M |
| --- | --- | --- | --- | --- | --- |
| 1 |  | 266.25 | 4.03 | 12.4 | 22.0 |
| 2 |  | 311.25 | 16.5 | >1000 | >1000 |
| 3 |  | 280.28 | 8.21 | 146 | 179 |
| 4 |  | 324.31 | 7.7 | 82 | 91 |
| 5 |  | 342.74 | 23.4 | >1000 | >1000 |
| 6 |  | 406.45 | 19.3 | >1000 | >1000 |
| 7 |  | 424.35 | 6.62 | 21.4 | 19.7 |
| 8 |  | 370.36 | 37.7 | 41.3 | 47.9 |
| 9 |  | 300.70 | 53.6 | 60.5 | 53.8 |
| 10 |  | 345.15 | 34.0 | 114.2 | 107.0 |
| 11 |  | 300.70 | 51.1 | >1000 | >1000 |
| 12 |  | 300.70 | 69.3 | >1000 | >1000 |
| 13 |  | 284.25 | 15.6 | 147.6 | 158.9 |
| 14 |  | 424.04 | 99.8 | 379.5 | 441.5 |
| 15 |  | 296.28 | 12.8 | 308.7 | 364.5 |
| 16 |  | 375.17 | 46.3 | 82.3 | 135.1 |
| 17 |  | 372.78 | 25 | 23 | 25 |
| 18 |  | 342.74 | 16.5 | 21 | 27 |
| 19 |  | 421.84 | 1.59 | 193.1 | 109.6 |
| 20 |  | 360.34 | 135.6 | 746.2 | 1017.6 |
| 24 |  | 407.42 | 51.3 | 75.8 | 100.0 |
| 25 | 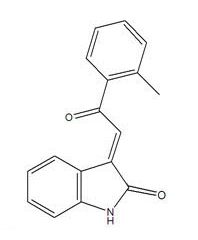 | 263 | 37.5 | 54.7 | 49.2 |
